# An Appeal to Parliament, the Medical Profession, and the Public, on the Present State of Dental Surgery

**Published:** 1841-12

**Authors:** 


*An Appeal to Parliament, the Medical Profession, and the Public, on the present state of Dental Surgery.* BY GEORGE WAITE, ESQ. Surgeon Dentist, M. R. C. S., &c. LONDON, HIGHLEY & Co. pp. 29, 8vo. 1841.

THE object of the author of this "appeal" is to obtain the passage of a law requiring of those exercising the duties of the dental surgeon, to undergo an examination before a Board, to be appointed by the Royal College of Surgeons. We heartily wish him success in this most laudable undertaking, and we are gratified to learn that our professional brethren in England are begining to be awakened to the importance of reform in this most important department of the curative art. We sincerely hope the respectable part of the profession there will cordially unite in this praiseworthy undertaking, and go about the matter in good earnest. Reform is loudly called for; the usefulness and respectability of the profession require it, and unless some test of qualification is instituted, the community must and will continue to suffer from the abuses which are at present so extensively practised. The efforts that are making in the United States, for the advancement of dental science, promise the most happy and salutary results, and we would suggest to our brethren in England, while attempting to better the condition of the profession there, by excluding from its practice all except such as are qualified, the propriety of furnishing the requisite facilities for instruction in those branches of knowledge, which it is essential for the dental surgeon to possess, to enable him to exercise the duties of his art in a skilful and scientific manner.—*Balt. Ed.*

